# SPREAD 4: online visualisation of pathogen phylogeographic reconstructions

**DOI:** 10.1093/ve/veac088

**Published:** 2022-09-26

**Authors:** Kanika D Nahata, Filip Bielejec, Juan Monetta, Simon Dellicour, Andrew Rambaut, Marc A Suchard, Guy Baele, Philippe Lemey

**Affiliations:** Department of Microbiology, Immunology and Transplantation, Rega Institute, KU Leuven, Herestraat 49, Leuven 3000, Belgium; Nonce Filip Bielejec, Łódź Voivodeship, 90-245 Lodz, Poland; Departamento de Montevideo, Guayabos 1924, Montevideo 11200, Uruguay; Department of Microbiology, Immunology and Transplantation, Rega Institute, KU Leuven, Herestraat 49, Leuven 3000, Belgium; Spatial Epidemiology Lab (SpELL), Université Libre de Bruxelles, CP160/12, 50 av. FD Roosevelt, Bruxelles 1050, Belgium; Institute of Evolutionary Biology, University of Edinburgh, Kings Building, Charlotte Auerbach Road, Edinburgh EH9 3FL, UK; Department of Human Genetics, David Geffen School of Medicine, University of California, 10833 Le Conte Ave, Los Angeles, CA 90095, USA; Department of Biostatistics, Jonathan and Karin Fielding School of Public Health, University of California, 650 Charles E Young Dr S, Los Angeles, CA 90095, USA; Department of Biomathematics, David Geffen School of Medicine, University of California, 10833 Le Conte Ave, Los Angeles, CA 90095, USA; Department of Microbiology, Immunology and Transplantation, Rega Institute, KU Leuven, Herestraat 49, Leuven 3000, Belgium; Department of Microbiology, Immunology and Transplantation, Rega Institute, KU Leuven, Herestraat 49, Leuven 3000, Belgium

**Keywords:** phylogeography, viral spread, BEAST, Bayesian inference, visualisation

## Abstract

Phylogeographic analyses aim to extract information about pathogen spread from genomic data, and visualising spatio-temporal reconstructions is a key aspect of this process. Here we present SPREAD 4, a feature-rich web-based application that visualises estimates of pathogen dispersal resulting from Bayesian phylogeographic inference using BEAST on a geographic map, offering zoom-and-filter functionality and smooth animation over time. SPREAD 4 takes as input phylogenies with both discrete and continuous location annotation and offers customised visualisation as well as generation of publication-ready figures. SPREAD 4 now features account-based storage and easy sharing of visualisations by means of unique web addresses. SPREAD 4 is intuitive to use and is available online at https://spreadviz.org, with an accompanying web page containing answers to frequently asked questions at https://beast.community/spread4.

## 1. Introduction

Genomic data with associated information about location and time of sampling offer opportunities to reconstruct how pathogens have spread through time and space. Both heuristic and model-based phylogeographic approaches have been developed for this purpose, and different types of phylogeographic models have been made available in order to tackle key questions on the emergence and spatial spread of infectious pathogens (Baele et al., 2018). Such phylogeographic inference methods, using both discrete and continuous location data, have become widespread in the field of pathogen phylodynamics (Grenfell et al., 2004) and have offered insights into the evolution and spread of various pathogens (Baele et al., 2017). These methods are available in a number of widely used phylogenetic and phylodynamic software packages (e.g. Suchard et al., 2018; Bouckaert et al., 2019; Sagulenko et al., 2018), with Bayesian inference approaches having greatly contributed to their popularity.

Considerable effort has been invested in improving phylogeographic models and associated statistical inference machinery (De Maio et al., 2015; Kühnert et al., 2016; Jackson et al., 2017; Müller et al., 2017; Guindon and De Maio, 2021; Hong et al., 2021), but practitioners remain confronted with the challenge of summarising and interpreting potentially complex estimation results. These results are typically visualised using a phylogenetic tree with leaves representing sampled observations and internal nodes together with connecting branches representing the inferred ancestral information (Revell, 2012; Yu et al., 2017). The growing popularity of phylogeographic models has called for better visualisation tools that can project pathogen spread on a geographic map, sometimes with interactive animations of the reconstructed evolution and spread over time (Theys et al., 2019). Feature-rich visualisation tools do justice to inferences from phylogeographic models as they make interpretation possible for a wide audience, which can help increase awareness and ultimately even motivate precautionary actions and inform public health agencies and policy-making authorities.

Data visualisation has embraced interactive web-based visualisation because it allows generating informative and reproducible interactive graphics that can be easily shared and exported to vector-based image formats, often using open-source software. Popular languages and packages include weave (web-based analysis and visualisation environment), plotly, and shiny (Sievert, 2020). Such web-based technologies have also permeated the research fields of phylogenetic and phylodynamic inference, and several popular tools have emerged in the past decade such as Evolview (He et al., 2016), iTol (Letunic and Bork, 2019), Microreact (Argimón et al., 2016), and Nextstrain (Aksamentov et al., 2021). These tools allow for easily shareable visualisations that can often be interpreted by scientific as well as non-scientific audiences alike (Theys et al., 2019). Such shareable visualisations aid the dissemination of scientific information and target the sense of curiosity (Bernasconi and Grandi, 2021).

Owing to their flexibility in visualizing analyses for a wide range of pathogens and the ease with which the results can be shared via social media, Nextstrain (Hadfield et al., 2018) and Nextclade (Aksamentov et al., 2021) have become primary examples that achieved broad visibility during the COVID-19 pandemic caused by the severe acute respiratory syndrome coronavirus 2 virus (SARS-CoV-2). The Nextstrain package offers a pipeline that combines data collection, phylogenetic analysis, and visualisation of the resulting trees as well as dedicated post-processing packages that focus on the visualisation task.

Here, we focus on the development of SPREAD (Bielejec et al., 2011; Bielejec et al., 2016), which aims at visualising the outcome of Bayesian phylogeographic inference, a process that is more time-consuming and less amenable to pipeline implementations. The first version of SPREAD (Bielejec et al., 2011) made use of Keyhole Markup Language, an Extensible Markup Language for expressing geographic annotation, in order to generate interactive visualisations in the Google Earth software package (http://earth.google.com). A first step towards browser-based visualisation and thereby avoiding the need to install custom software packages was made by spreaD3 (Bielejec et al., 2016). SpreaD3 used data-driven documents (i.e. JavaScript D3 libraries) and required the user to operate a standalone Graphical User Interface for parsing the files as well as a web browser for the final visualisation, making the entire process cumbersome. The output of previous versions was also not easily shareable with other researchers or on social media. Finally, a series of updates to commonly used browser platforms have now necessitated an overhaul of spreaD3 to maintain its ease of use for a wide range of users.

## 2. Approach

SPREAD 4 is an online visualisation tool that allows users to sign in with their e-mail address or with their Google account to access its functionalities. The new version stores the user’s visualisations on the web server, allowing to revisit or share the analysis results with others. Upon uploading an annotated phylogeny, SPREAD 4 parses it in three steps: ongoing data analysis, queued or completed data analysis. At the ongoing data analysis stage, the user is asked to complete the settings or any additional requirements for visualisation such as selecting the annotation representing longitude and latitude in a continuous phylogeographic reconstruction or setting the most recent sampling date and time multiplier (in case the timescale is not in years). After this stage, the analysis is queued and when completed, the user can choose to visualise the reconstruction on the default world map or upload a custom geoJSON file that holds a particular region of interest. If the user chooses to visualise on the default world map, SPREAD 4 automatically retrieves more fine-grained geographic resolution for the area of the world map on which most of the transitions occur while keeping a basic map for the rest of the world.

The visualisation consists of an animation over time and is associated with a shareable web address (Uniform Resource Locator; URL) that can be copied and opened in any desired browser on any operating system, ensuring reproducibility and transparency. For each uploaded data set, the user can choose to visualise the entire phylogeographic history unfolding over time or render transitions or dispersal events as ‘missiles’ from one location to another. This new feature of SPREAD, motivated by Dudas (2022), provides a filtered view of the transition history emphasising the transitions occurring at any particular time point. In addition to this feature, the user can choose to visualise the transitions using a colour gradient determined by an annotated attribute such as the posterior median rate (default) height or length, posterior probabilities, etc. Furthermore, the user can choose to filter the animation according to attribute values such as only visualising nodes with a posterior probability within a specified range.

At any point in time, the user can click on any object in the map to retrieve more details such as the posterior modal location state and its probability at a particular node in a discrete phylogeographic reconstruction or the node support. The visualisations can also be exported as Scalable Vector Graphics files that can be used as publication-ready figures.

SPREAD 4 typically visualises output files containing trees annotated with discrete or continuous locations and is therefore primarily designed for use in conjunction with the BEAST software package (Suchard et al., 2018; Bouckaert et al., 2019). However, SPREAD 4 can also process output files generated by other phylogenetic and phylodynamic inference applications, as long as the nodes and branches are annotated using a compatible syntax. Three different types of input files—corresponding to output from three different types of analyses (Sections 2.1, 2.2 and 2.3)—can be visualised in SPREAD 4, with its main use being the visualisation of a maximum clade credibility (MCC) tree (https://beast.community/summarizing_trees) summarising either a discrete or continuous phylogeographic analysis.

### 2.1 Discrete phylogeography: MCC tree

**Figure 1.**
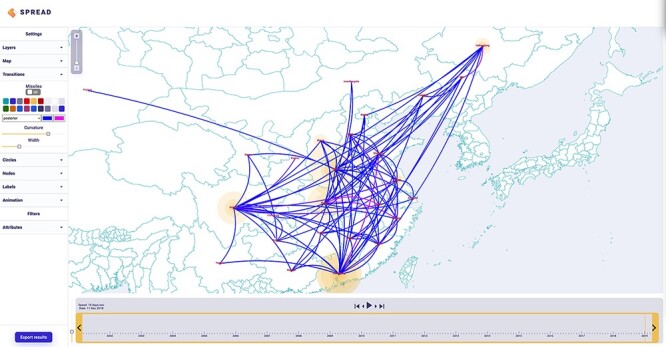
Discrete phylogeographic transition history of porcine epidemic diarrhoea virus (He et al., 2022) in China. A color gradient reflects the time of the estimated transition events.

Sequences are often associated with discrete sampling locations, such as a municipality, district, province, or country. SPREAD 4 associates user-provided geographic coordinates to these locations to map transitions between them over a time interval determined by the branch length estimates. Location transitions are represented by lines or missiles on the map, while branches maintaining a location state are visualised using customised circular polygons (see Fig. 1). Starting from the inferred time of origin until the most recent sample in the data set, SPREAD 4 offers a timeline view of the ancestral reconstruction on a geographic map. This map can be easily customised based on user preferences. For example, the user can select which layers to show, alter the colours or widths of the transitions, and alter the colours of the circles, nodes, and labels shown.

### 2.2 Discrete phylogeography: transition rate support

**Figure 2.**
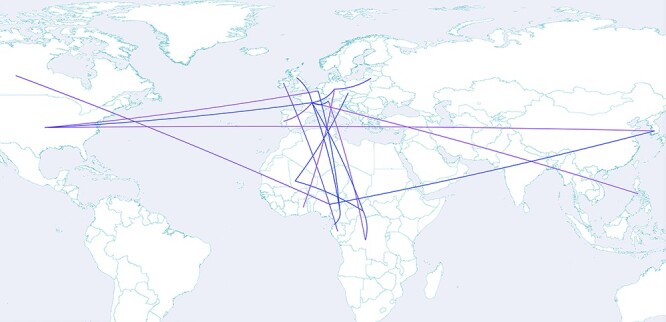
Discrete transition rates between countries with a posterior probability support >0.5 for SARS-CoV-2 lineage B.1.620 (Dudas et al., 2021). A color gradient reflects the Bayes factor support for the transition rates.

Discrete phylogeographic inference attempts to estimate transition rates between all pairs of sampled locations, potentially involving a large number of pairwise rate parameters. Most genomic data sets are unlikely to contain information about all possible transitions for large-dimensional problems. Poorly informed rate estimates could lead to high variance estimates for the inferred ancestral locations. To address this problem, Lemey et al. (2009) introduced Bayesian stochastic search variable selection, which allows selecting a sparse set of parameters to be estimated based on their support from the data. This support can be expressed by means of a posterior rate indicator expectation or a ‘Bayes factors’ value and visualised in SPREAD 4 as a network plot of the supported transitions on a geographic map. Here, we show an example for the supported migration rates in the geographic spread of SARS-CoV-2 lineage B.1.620 (see Fig. 2) (Dudas et al., 2021). The user can customise this map to show the transition colours as a gradient that reflects the respective support value.

### 2.3 Continuous phylogeography: MCC tree

**Figure 3.**
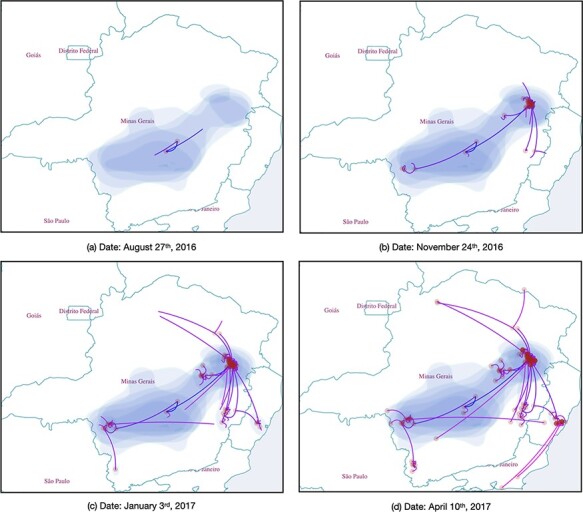
Continuous phylogeographic transition history of yellow fever virus across western Brazil (Faria et al., 2018) over four different time points. A color gradient reflects the time of the estimated dispersal events.

While discrete phylogeographic inference uses information from potentially large discrete areas such as entire countries, continuous phylogeographic inference (Lemey et al., 2010) is able to use more fine-grained location data such as longitude and latitude coordinates. Acquiring such detailed location information can be challenging (Dellicour et al., 2022) but very useful for generating highly detailed spatio-temporal patterns of spread. Such analyses have also been used to quantify the rate of spread and its heterogeneity (Pybus et al., 2012), to test hypothetical intervention strategies (Dellicour et al., 2018), or to investigate the impact of environmental factors on the dispersal of viruses (Dellicour et al., 2020). Similar to visualising a discrete phylogeographic inference outcome, SPREAD 4 shows dispersal events using lines or missiles on a geographic map, but also plots the uncertainty of geographic coordinates at the internal nodes through their annotated highest posterior density contours (see Fig. 3).

## Data Availability

SPREAD 4 is an open-source software under the MIT License and can be accessed at https://spreadviz.org/. Its source code is available at https://github.com/phylogeography/spread for further software development or compiling the latest custom build. While the user interface and features of SPREAD are very intuitive, a web page with the most frequently asked questions can be found at https://beast.community/spread4.
